# Clinical Evaluation of NESOSPRAY HE-C, a Nasal Spray, for Rhinopharyngitis and Rhinosinusitis: A Randomized, Double-Blind, Placebo-Controlled Trial

**DOI:** 10.3390/medicina61061071

**Published:** 2025-06-11

**Authors:** Fatima-Zahra El Barche, Manon D’almeida, Séverine Dameron, Rémi Shrivastava

**Affiliations:** Vitrobio Research Institute, ZAC de Lavaur, 63500 Issoire, France

**Keywords:** rhinopharyngitis, rhinosinusitis, nasal spray, medical device

## Abstract

*Background and Objectives*: The common cold (acute rhinopharyngitis) and acute rhinosinusitis are highly prevalent conditions that significantly impact quality of life, often leading to nasal congestion, inflammation, and discomfort. Given the growing demand for non-pharmacological treatment options, particularly for vulnerable populations such as children and pregnant women, alternative therapies are increasingly being explored. NESOSPRAY HE-C, a nasal spray formulated with a glycerol-based filmogenic solution, acts by forming a protective osmotic film on the nasal mucosa. This mechanism facilitates mechanical cleansing, enhances decongestion, and reduces inflammation while preserving mucosal integrity. Its purely topical and mechanical mode of action provides a non-systemic alternative for symptom management. *Materials and Methods*: This randomized, double-blind, parallel-group clinical trial evaluated the efficacy and safety of NESOSPRAY HE-C (*n* = 29) compared to a placebo nasal spray (*n* = 26) in patients aged ≥ 3 years diagnosed with the common cold or acute rhinosinusitis. Participants had a baseline Rhinosinusitis Symptom Severity Score (RSSS) of ≥25/50. Treatment consisted of administering 2–3 sprays per nostril, four times daily, every 4 to 6 h, for up to 8 days or until symptom resolution. The primary outcomes included changes in total RSSS, Wisconsin Upper Respiratory Symptom Survey (WURSS) score, and individual symptom scores (rhinorrhea, nasal congestion, cough, poor sleep, facial pain, and fever). Safety assessments included adverse event monitoring and treatment tolerability, with subgroup analyses performed for children and pregnant women. *Results*: Baseline demographics were comparable between the treatment groups. NESOSPRAY HE-C demonstrated a significantly greater reduction in total RSSS from Day 3 onward (*p* = 0.0008), with sustained superiority through Day 8 (*p* < 0.0001). Significant improvements in rhinorrhea and nasal congestion were observed within 2 h of administration (*p* = 0.0089), while reductions in cough (*p* = 0.0052), poor sleep (*p* = 0.0005), and facial pain (*p* = 0.0111) emerged by Day 3. Fever reduction was most pronounced on Days 6 (*p* = 0.0001) and 8 (*p* = 0.0312), indicating a delayed but significant effect. In terms of the WURSS score, NESOSPRAY HE-C showed a significant improvement from Day 1, with a greater reduction in symptom severity compared to placebo. This trend of greater improvement continued through Day 8. The treatment was well tolerated, with no reports of serious adverse events or allergic reactions. Efficacy was consistent across all subgroups, including children, pregnant women, and adults. *Conclusions*: NESOSPRAY HE-C provides rapid and sustained symptom relief for the common cold and acute rhinosinusitis, serving as a safe and effective non-pharmacological alternative to conventional treatments. By leveraging its osmotic action and barrier-forming properties, it facilitates mechanical cleansing, enhances decongestion, and reduces inflammation while preserving mucosal integrity. Additionally, by forming a protective film on the nasal mucosa, it protects against future irritations, further supporting its role as a valuable therapeutic option, particularly for individuals seeking non-systemic symptom management.

## 1. Introduction

Both rhinopharyngitis (better known as the common cold) and rhinosinusitis are prevalent upper respiratory conditions that significantly impact quality of life [1,2]. The common cold, primarily caused by viral infections, is characterized by symptoms such as nasal congestion, rhinorrhea, cough, and mild systemic signs [3]. It affects millions of individuals worldwide annually [4]. Notably, the common cold often precedes the onset of rhinosinusitis [1]. In contrast, rhinosinusitis is an inflammation of the nasal passages and paranasal sinuses [5], often resulting from viral infections, although it can also be triggered by bacterial infections, allergies, or anatomical variations [6]. Approximately 12% of the population is affected by rhinosinusitis each year [5], leading to both direct medical costs and indirect costs such as lost productivity.

In Europe, the cost per acute rhinosinusitis (ARS) episode was estimated at around EUR 1100 in 2020 [6]. Rhinosinusitis can be classified into acute, subacute, chronic, and recurrent types based on the duration and frequency of symptoms. ARS lasts less than four weeks [7,8], subacute rhinosinusitis lasts between four to twelve weeks, chronic rhinosinusitis (CRS) persists for more than twelve weeks, and recurrent rhinosinusitis involves multiple episodes within a year [8,9]. Both conditions share similar symptoms such as nasal congestion and facial discomfort, with rhinosinusitis often presenting with more pronounced facial pain and purulent discharge [9]. The pathophysiology of these conditions involves the obstruction of sinus drainage pathways and the accumulation of mucus, which can lead to infection and inflammation. Factors such as viral infections, bacterial infections, and environmental triggers play a significant role in the onset and progression of both conditions [10].

- Considerations for Pregnant Women

Pregnant women with the common cold or rhinosinusitis require special consideration due to potential risks to both the mother and fetus. Hormonal changes during pregnancy can worsen nasal congestion and inflammation, further complicating symptom management [11]. Treating these conditions in pregnant patients is particularly challenging, as many pharmacological options are restricted to minimize potential risks to fetal development. Therefore, non-pharmacological interventions and safe, localized treatments that provide effective symptom relief without systemic exposure are essential for ensuring both maternal well-being and fetal safety [12].

- Considerations for Pediatric Population

Due to their immature immune systems and narrower nasal passages, pediatric patients are more susceptible to complications, including otitis media, orbital cellulitis, and, in rare cases, intracranial infections [13]. Effective management should prioritize symptom relief, maintaining nasal hygiene, and reducing the risk of complications, while ensuring that treatments are age-appropriate and safe. Given the limited pharmacological options available for young children, non-pharmacological interventions and well-tolerated treatments play a crucial role in managing these conditions.

- NESOSPRAY HE-C as a Non-Pharmacological Intervention

Non-pharmacological treatments such as isotonic or hypertonic saline nasal irrigation, steam inhalation, and barrier-forming nasal gels or sprays are commonly used to relieve nasal symptoms [14,15,16]. While these approaches improve mucociliary clearance and moisturize the nasal mucosa, their effects may be short-lived or inconsistent, especially in moderate-to-severe cases. In contrast, NESOSPRAY HE-C combines high osmotic activity for rapid decongestion with a long-acting filmogenic barrier that protects the nasal epithelium, offering both immediate and sustained symptom relief without systemic exposure. NESOSPRAY HE-C is an internationally patented nasal spray device formulated with a specialized filmogenic glycerol that integrates natural polymers derived from plant extracts [17]. This innovative formulation offers a novel, non-pharmacological approach to managing conditions such as the common cold (rhinopharyngitis) and rhinosinusitis. Its high osmotic capacity—up to ten times greater than that of seawater—enables the mechanical removal of fluids and contaminants from the inflamed nasal mucosa while simultaneously forming a stable protective film that persists for several hours [18]. This dual action helps reduce inflammation and protect the nasal passages from external irritants, making NESOSPRAY HE-C particularly beneficial for populations in whom systemic treatments are contraindicated or should be avoided. Importantly, its action is purely topical, mechanical, and localized within the nasal cavity, with no systemic activity.

- Objectives of the Present Study

This study aims to evaluate the efficacy and safety of NESOSPRAY HE-C in comparison to a placebo for the treatment of the common cold and ARS in diverse populations, including children and pregnant women. The primary objective is to assess improvements in symptoms such as rhinorrhea, nasal congestion, cough, sleep quality, and facial pain. Secondary objectives include the evaluation of tolerability and the safety profile of NESOSPRAY HE-C.

## 2. Methods

### 2.1. Study Design and Ethics

This study was designed as a comparative, randomized, double-blind, parallel-group interventional clinical trial. It was conducted in compliance with the International Council for Harmonisation (ICH) Good Clinical Practice (GCP) guidelines and Indian GCP standards. The trial was registered under the Clinical Trials Registry—India (CTRI) with the registration number CTRI/2024/06/062329 on 25/06/2024. Ethical approval was obtained from the relevant independent ethics committee, and written informed consent was provided by all participants or their legal guardians before enrollment. The study was sponsored by Polytrap Pharma Pvt. Ltd. (Indore, India), and operationally managed by Mudra Clincare, Navi Mumbai, India.

### 2.2. Study Population

#### 2.2.1. Inclusion Criteria

Participants included male and female patients aged ≥ 3 years with a clinical diagnosis of upper respiratory infection, either from the common cold or acute rhinosinusitis (ARS), and a baseline Rhinosinusitis Symptom Severity Score (RSSS) ≥ 25/50. The RSSS is a validated tool that quantifies disease burden by assessing key symptoms such as nasal congestion, rhinorrhea, facial pain or pressure, cough, and sleep disturbance. Both adults and children were eligible, with pregnant women over 18 years included if they met all criteria. Children (3–18 years) were required to be accompanied by a parent or caregiver. Participants agreed to refrain from medications that could influence study outcomes, and if their condition worsened, study physicians could prescribe necessary treatments. The use of saline nasal sprays, nasal lavages, eye drops, or herbal treatments was also prohibited during the study period.

#### 2.2.2. Exclusion Criteria

Patients were excluded if they had hypersensitivity to any investigational product component, were younger than 3 years, or had conditions impairing breathing, such as bronchopneumonia. Additional exclusions included a history of recent nasal surgery, chronic allergy treatments, immunosuppression, or recent use (within two weeks prior to screening) of antihistamines, steroids, antibiotics, or antiviral treatments. Patients with structural abnormalities affecting the sinuses (e.g., deviated septum, nasal polyps) or those who had participated in another clinical trial within the past year were also excluded. Eligible participants meeting all inclusion and no exclusion criteria were enrolled and randomized to receive either the test or reference product.

### 2.3. Special Considerations for Pediatric and Pregnant Populations

Children included in this study participated with the written informed consent of their parents or legal guardians. Parents were provided with comprehensive instructions on how to administer the investigational product and were responsible for ensuring their child’s adherence to the prescribed treatment regimen. A pediatrician closely monitored the children throughout the trial, conducting safety evaluations and assessing symptoms.

Pregnant women were eligible to participate only if they were in their first or second trimester, in order to minimize potential risks.

### 2.4. Intervention and Randomization

Patients were randomized in a 1:1 ratio to receive either the investigational product, Nasal Spray HE-C, or a placebo comparator spray. Randomization was conducted using SAS software (Version 9.1.3) with block randomization to ensure balanced allocation within predefined age groups (3–18 years and >18 years). Blinding was maintained for all investigators, patients, and caregivers regarding treatment assignments.

The investigational product, NESOSPRAY HE-C, is a nasal spray comprising glycerol, water, a combination of Acacia gum and Xanthan gum, extracts of Ribes nigrum fruit and Curcuma longa rhizome, essential oils of Mentha piperita, Eucalyptus globulus, Thymus satureioides, and Rosmarinus officinalis, as well as potassium sorbate, sodium benzoate, and citric acid. Its mechanism of action is mechanical, producing an osmotic decongestant effect to reduce mucosal edema, forming a protective filmogenic barrier on the nasal mucosa, and facilitating the cleansing of nasal passages.

The placebo comparator was a thickened aqueous solution designed to mimic the physical properties of glycerol-based spray, including density, appearance, packaging, and administration method. It contained water, Solagum (a blend of Acacia gum and Xanthan gum), potassium sorbate, and sodium benzoate.

Both sprays were supplied in 15 mL containers equipped with a spray applicator. The recommended administration was 2–3 sprays per nostril, four times daily, at intervals of approximately 4–6 h.

### 2.5. Study Procedures and Assessments

After confirming eligibility during the screening process, baseline data were collected, including demographic information, vital signs, physical examination findings, the Rhinosinusitis Symptom Severity Score (RSSS), and the Wisconsin Upper Respiratory Symptom Survey (WURSS-21) score.

The RSSS is a composite clinical score designed to quantify the overall severity of rhinosinusitis symptoms. It is calculated by summing the severity scores (each rated from 0 to 10) of five core symptoms: rhinorrhea or nasal congestion, cough, sleep disturbance, facial pain or pressure, and fever. The total RSSS ranges from 0 to 50, with higher scores indicating greater symptom severity. These five symptoms were also analyzed individually as individual symptom scores to monitor the progression of each symptom over time.

The WURSS-21 [19,20,21] is a validated, patient-reported outcome tool used to assess the severity and functional impact of upper respiratory tract infections. It includes 20 scored items: 10 related to symptom severity (e.g., runny nose, sneezing, sore throat, nasal congestion, cough, feeling tired), and 9 assessing functional impairments (e.g., sleep quality, ability to think clearly, and to accomplish daily activities), each rated on a 7-point Likert scale. One additional item assesses global change in illness. The total WURSS score is computed by summing the first 20 items, with higher scores reflecting greater overall disease burden. Several of the individual symptom scores (e.g., nasal congestion, cough, fatigue, and sleep) from the WURSS overlap with those included in the RSSS and were analyzed in parallel.

Study visits and symptom assessments were conducted at baseline (Day 0), 2 h after the initial dose, and Day 1 (24 h), Day 3, Day 6, and Day 8 or upon earlier recovery. Adherence to the dosing regimen was monitored at each visit through patient or caregiver reports and investigator questioning. For pediatric participants, parents or guardians were instructed to administer the treatment and report usage accuracy. At each assessment point, symptom severity was evaluated by participants or, in the case of younger patients, their parents or caregivers. The primary endpoints were changes in the overall RSSS and key symptoms, including rhinorrhea/congestion, fever, cough, poor sleep quality, and facial pressure pain. Additionally, the study evaluated the overall severity of cold symptoms and their impact on participants’ quality of life by tracking the total WURSS score over time. Secondary endpoints included changes in individual symptom scores, global assessments by participants, parents, and physicians at the study’s conclusion, as well as ratings of product acceptability. Adverse events (AEs) and serious adverse events (SAEs) were closely monitored throughout the study.

### 2.6. Blinding and Unblinding

The investigational and placebo products were indistinguishable in appearance, packaging, and labeling, with unique product codes accessible exclusively to the sponsor’s designated personnel. Patients, investigators, and clinical staff were blinded to treatment allocation until the database was locked. Emergency unblinding was permitted when required for patient safety, with each unblinding event thoroughly documented and justified.

### 2.7. Statistical Analysis

Based on prior clinical data from studies involving similar glycerol-based nasal sprays, an effect size was estimated. Assuming a two-sided significance level (α) of 0.05, a statistical power of 80% (1–β), and accounting for an anticipated dropout rate of 10%, the required sample size was determined to be 60 patients (30 per treatment arm). To strengthen the robustness of the analysis, 65 patients were ultimately enrolled. Of these, 55 participants completed the study and were included in the final analysis (n = 29 in the NESOSPRAY HE-C group, n = 26 in the placebo group). The analysis set included participants who attended all study visits and completed all required assessments. Baseline demographic and clinical characteristics were compared between the treatment groups using appropriate statistical tests, including Student’s *t*-test, Wilcoxon rank-sum test, and Fisher’s exact test, to confirm population homogeneity. Efficacy endpoints were analyzed using two-way ANOVA with interaction in mixed linear models, followed by post hoc tests (Student’s *t*-test or Wilcoxon rank-sum test) for pairwise comparisons at each time point. The frequency of adverse events (AEs) was assessed using Chi-square tests. While the study population included children, adults, and pregnant women, primary analyses were conducted on the full cohort. Secondary analyses confirmed that trends and treatment effects were consistent across these subpopulations.

## 3. Results

### 3.1. Whole Population

A total of 55 patients were included in the efficacy and safety analysis, with 29 patients receiving NESOSPRAY HE-C and 26 patients receiving the placebo comparator. Baseline demographic characteristics, including the age and sex distribution of the overall population, were well-balanced between the two groups as summarized in Table 1.

### 3.2. Pediatric Subgroup Analysis

A total of 19 pediatric patients (aged ≤ 17 years) were included in the study, representing 34.5% of the overall population. Among them, 10 children (53%) received the placebo, while 9 children (47%) were treated with NESOSPRAY HE-C. Sex distribution was comparable between the two groups (*p* = 1.00). The mean age in this subgroup was 10.6 ± 3.6 years, with no significant difference between treatment groups (*p* = 0.36). Detailed demographic data for the pediatric population are presented in Table 2.

### 3.3. Pregnant Women Subgroup Analysis

Seventeen pregnant women were included in the study, representing 30.9% of the overall population. Of these, 7 patients (41%) received the placebo, while 10 patients (59%) received NESOSPRAY HE-C. The mean age in this subgroup was 30.1 ± 3.2 years, with no statistically significant difference between groups (*p* = 0.70). Detailed demographic characteristics are outlined in Table 3.

### 3.4. Primary Endpoint

#### 3.4.1. Rhinosinusitis Symptom Severity Score (RSSS)

**Figure 1:**
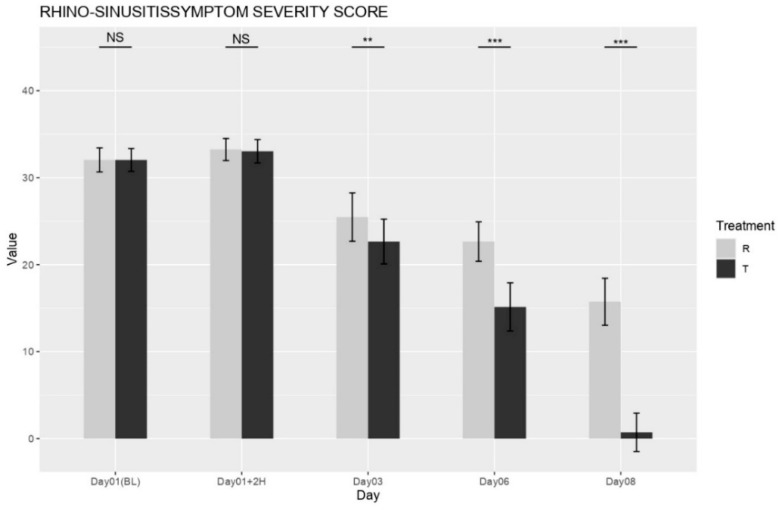
Changes in the total RSSS between the treated group (T) and the placebo group (R) over time. NS = non-significant, ** = *p* < 0.001, *** = *p* < 0.0001.

The mean baseline Rhinosinusitis Symptom Severity Score (RSSS; 0–50 scale) did not significantly differ between the NESOSPRAY HE-C and placebo groups (*p* = 0.4532), ensuring an equal distribution of disease severity at study initiation. Beginning on Day 3, the NESOSPRAY HE-C group (Treatment T) exhibited a statistically significant reduction in total RSSS compared to the placebo group (Treatment R) as shown in Figure 1. On Day 3, the between-group difference in total RSSS was highly significant (*p* < 0.001). By Day 6, this difference became even more pronounced (*p* < 0.0001), indicating a substantial and sustained improvement in symptom severity. These findings support the conclusion that NESOSPRAY HE-C provides progressive and durable symptom relief.

#### 3.4.2. Wisconsin Upper Respiratory Symptom Survey (WURSS) Score

**Figure 2:**
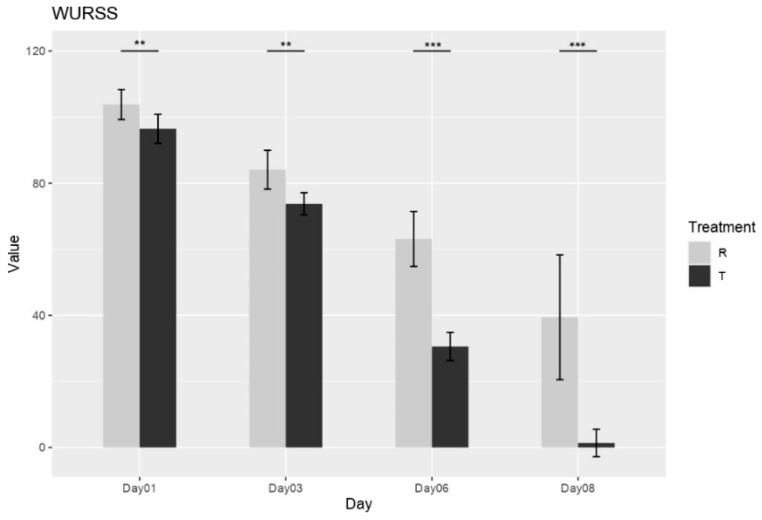
WURSS-21 mean scores, assessed for both R and T Groups at Day 1, and on subsequent follow-up days (Days 3, 6, and 8). ** = *p* < 0.001, *** = *p* < 0.0001.

The influence of treatment on upper respiratory symptom severity was measured by WURSS score. Immediately following the first dose (Day 1), a significant difference in symptom burden was already apparent, with Group R exhibiting a higher mean WURSS-21 score (103.73 ± 4.36) compared to Group T (96.63 ± 4.42). This difference favoring Group T persisted and became more pronounced throughout the study duration. By Day 3, both groups showed a reduction in symptom scores; however, the improvement was notably greater in Group T (73.87 ± 3.34) compared to Group R (83.96 ± 5.79). This trend of greater symptom reduction in Group T continued, becoming increasingly evident on Day 6 (Group T: 30.55 ± 4.26; Group R: 63.12 ± 8.31) and Day 8 (Group T: 1.38 ± 4.15; Group R: 39.42 ± 18.88) (see Figure 2).

### 3.5. Individual Symptom Outcomes (Secondary Endpoints)

**Figure 3:**
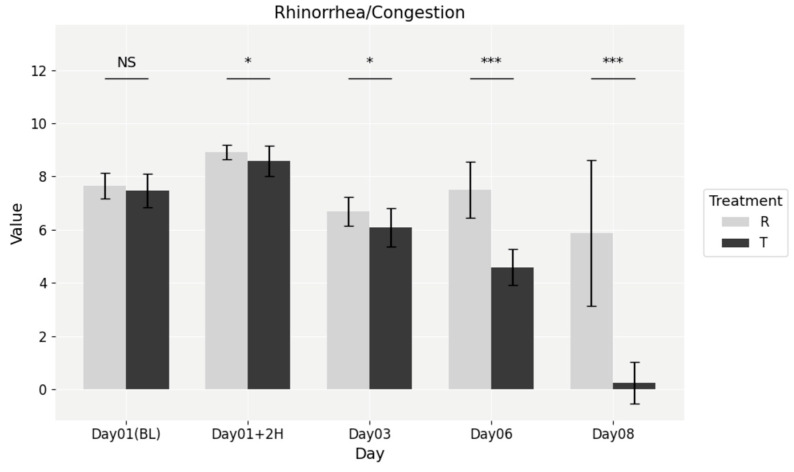
Temporal evolution of rhinorrhea/congestion symptom scores. NS = non-significant, * = *p* < 0.01, *** = *p* < 0.0001.

1. Nasal Congestion (Rhinorrhea/Congestion):

At baseline, no significant differences were observed between the NESOSPRAY HE-C and placebo groups (*p* = 0.3557). However, by the 2 h post-dose mark, a statistically significant difference emerged (*p* = 0.0088), favoring NESOSPRAY HE-C. This difference persisted through subsequent time points, as shown in Figure 3, including Day 3 (*p* = 0.0030), Day 6 (*p* = 1.381 × 10^−8^), and Day 8 (*p* = 7.554 × 10^−9^). Treatment with NESOSPRAY HE-C resulted in a faster and more pronounced reduction in nasal congestion, leading to improved nasal airflow and overall comfort.

2. Cough:

No significant differences were observed between the treatment groups at baseline (*p* = 0.247) or 2 h post-dose (*p* = 0.787). However, by Day 3, the NESOSPRAY HE-C group demonstrated a statistically significant improvement (*p* = 0.0052), which remained highly significant at Day 6 (*p* = 1.00 × 10^−^⁷) and Day 8 (*p* = 8.43 × 10^−^⁹). These findings suggest a robust and sustained reduction in cough frequency and severity in the NESOSPRAY HE-C group.

3. Sleep Quality:

While a significant baseline difference was observed (*p* = 0.04933), by Day 3, the NESOSPRAY HE-C group exhibited a highly significant improvement (*p* = 0.0005) in sleep quality. This benefit persisted through Day 6 (*p* = 7.664 × 10^−^⁶) and Day 8 (*p* = 7.012 × 10^−^⁹), emphasizing the effectiveness of NESOSPRAY HE-C in enhancing sleep over time.

4. Pain Upon Facial Pressure:

Pain scores were comparable at baseline (*p* = 0.761). However, by Day 3, a significant reduction in facial pain was observed in the NESOSPRAY HE-C group (*p* = 0.0111), with further sustained improvement through Day 6 (*p* = 1.01 × 10^−^⁴) and Day 8 (*p* = 1.25 × 10^−^⁷). These findings indicate that NESOSPRAY HE-C provides effective and prolonged relief from sinus-related pressure and discomfort.

5. Fever:

In the treatment group, 26 out of 29 patients (89.7%) presented with fever on Day 1. This number remained unchanged at 2 h post-dose, decreased slightly to 24 patients on Day 3 and Day 6 (82.8%), and dropped to 0 by Day 8. In the placebo group, 23 out of 26 patients (88.5%) had fever at baseline, 24 (92.3%) after 2 h, 19 (73.1%) on Day 3, 16 (61.5%) on Day 6, and 4 patients (15.4%) on Day 8. These data show a notably faster and complete resolution of fever in the NESOSPRAY HE-C group compared to placebo, particularly evident by Day 8. The difference between groups was statistically significant on Day 6 (*p* = 0.00014) and Day 8 (*p* = 0.0312).

These results underscore the consistent and significant improvement observed in patients treated with NESOSPRAY HE-C across multiple symptom domains, particularly in nasal congestion, cough, sleep quality, facial pain, and fever reduction (Table 4).

The temporal pattern of symptom relief observed in this study provides important insights into the mode of action and clinical relevance of NESOSPRAY HE-C. The early and significant reduction in nasal congestion within 2 h post-administration supports the proposed osmotic decongestant mechanism, whereby fluid is rapidly drawn from the mucosal tissue to the surface, facilitating clearance of inflammatory exudates. This effect was not only rapid but sustained through Day 8, indicating that the protective barrier formed on the mucosa may also prevent further irritation or pathogen adhesion.

Improvements in other symptoms such as cough, sleep, and facial pain began by Day 3, a timeline consistent with downstream effects of reduced postnasal drip and mucosal inflammation. Fever resolution was more gradual, with significant differences between groups becoming apparent by Day 6. This delayed response suggests that while NESOSPRAY HE-C does not have a systemic antipyretic effect, its local action may contribute to reducing the inflammatory load in the upper airways over time.

### 3.6. Global Assessments

Global assessments performed by patients/parents and physicians on Day 8 or at the time of recovery demonstrated a greater improvement in the NESOSPRAY HE-C group compared to the placebo group. Specifically, 26 participants in the NESOSPRAY HE-C group were rated as “very much improved”, while 3 participants were rated as “much improved”. In contrast, the placebo group had only 4 participants rated as “very much improved”, while 3 participants were rated as “much improved”, and a significant proportion (19 participants) were rated as “minimally improved”. The detailed global assessment ratings are presented in Table 5.

### 3.7. Safety and Tolerability

Both treatments were well tolerated, and no serious adverse events were reported. All adverse events (AEs) were assessed through direct interviews at each follow-up visit (Days 1, 3, 6, and 8) and recorded using standardized case report forms. During the second visit, five patients reported AEs: two from the NESOSPRAY HE-C group (headache and loss of taste) and three from the placebo group (loss of smell, loss of taste, and dyspnea). At the third visit, one additional placebo-treated patient reported loss of smell, and another case of loss of taste was reported at the fourth visit, also in the placebo group. No allergic reactions occurred, and all AEs were transient, non-serious, and resolved without medical intervention. No statistically significant difference in the incidence of local irritation or other mild adverse effects was observed between groups (*p* = 0.8954). These safety findings were consistent across age groups and in pregnant participants.

### 3.8. Subgroup Consistency

The study included a diverse population comprising children (≥3 years), adults, and pregnant women, but no separate efficacy analysis was conducted for each subgroup. Instead, we assessed whether the treatment effect varied across subgroups by testing the interaction between treatment and subgroup using a repeated-measures approach applied to the entire population. The analysis revealed no significant interaction, suggesting that children and pregnant women exhibited similar symptom improvement patterns as the overall population. Specifically, pregnant participants in their first or second trimester (excluding the third trimester) experienced similar symptom relief in both extent and timing as non-pregnant adults (*p* > 0.05 for interaction). Similarly, children (≥3 years) demonstrated consistent symptom resolution trajectories, indicating that age did not influence the overall treatment effect (*p* > 0.05 for interaction).

Further evidence of consistency was provided by the absence of subgroup-specific safety concerns. The incidence of adverse events and the overall tolerability profile were comparable across all age groups and among pregnant participants, aligning with the total study population. Thus, although separate subgroup analyses were not required by the protocol, these findings support the conclusion that NESOSPRAY HE-C is both effective and safe for children, adults, and pregnant women.

## 4. Discussion

This randomized, double-blind, placebo-controlled clinical trial demonstrates that NESOSPRAY HE-C, a purely mechanical nasal spray, provides both rapid and sustained relief of symptoms associated with the common cold (acute rhinopharyngitis) and acute rhinosinusitis compared to a placebo. By Day 3, participants using NESOSPRAY HE-C showed significant improvements in key symptoms, as measured by the Rhinosinusitis Symptom Severity Score (RSSS). Similarly, the Wisconsin Upper Respiratory Symptom Survey (WURSS) score revealed a significant reduction in symptom severity in the treatment group, with a greater improvement observed from Day 1 through Day 8 compared to the placebo group. Notably, a rapid reduction in nasal congestion was observed within hours of administration, and these improvements were sustained through Day 8. Additionally, NESOSPRAY HE-C was effective in relieving several symptoms, such as facial pain, enhancing sleep quality, and reducing coughing potentially caused by postnasal drip. These findings suggest that the osmotic decongestant and protective barrier effects of NESOSPRAY HE-C facilitate quicker symptom relief during the acute phase of illness. The osmotic action of the glycerol-based film generates a local hyperosmotic gradient, drawing hypotonic fluid from the nasal epithelium toward the mucosal surface. This promotes the outward movement of fluids, which assists in the mechanical removal of mucus, inflammatory exudates, pathogens, and environmental irritants. Combined with the barrier-forming polymers that stabilize the film, this mechanism supports mucosal cleansing and protection, consistent with the pathophysiology and treatment principles of acute rhinosinusitis and the common cold.

The observed results align with existing clinical recommendations that favor non-pharmacological treatments for managing common cold and ARS, particularly in populations where pharmacological treatments might pose risks or be poorly tolerated. While certain intranasal corticosteroids are approved for use in children and pregnant women and have demonstrated safety profiles comparable to placebo, concerns about systemic absorption, long-term use, and clinical preference for drug-free alternatives in these populations still support the exploration of non-pharmacological treatments such as NESOSPRAY HE-C. The efficacy of NESOSPRAY HE-C across various demographic subsets, including children and pregnant women, underscores its potential as a universally applicable treatment. Moreover, the absence of serious adverse events or allergic reactions further supports the safety profile of NESOSPRAY HE-C, positioning it as a suitable option for first-line or adjunctive therapy.

It should be noted that this study did not include a comparator arm involving intranasal corticosteroids or antibiotics. As such, while NESOSPRAY HE-C demonstrated clear superiority over placebo in symptom relief, its efficacy relative to conventional pharmacological therapies remains to be established in future head-to-head studies. When compared to saline irrigations, NESOSPRAY HE-C’s dual osmotic and barrier effects may offer a clinically significant advantage. However, further studies with direct head-to-head comparisons against other non-pharmacological treatments are necessary to solidify these findings.

Despite the promising results, there are limitations to this study. The sample size, while sufficient to demonstrate statistically significant differences, was relatively small and focused primarily on short-term outcomes. Although the present study demonstrated clear symptomatic improvements using validated subjective scales, it did not include objective nasal airflow measurements such as Peak Nasal Inspiratory Flow (PNIF), which could have provided complementary data to support the observed effects. Future studies should incorporate PNIF to enhance the robustness of clinical evaluation. Long-term follow-up is essential to determine the duration of symptom relief, particularly in chronic rhinosinusitis (CRS). Additionally, although the study observed consistent trends across children, adults, and pregnant women, future research should investigate subgroup-specific outcomes in larger, more diverse populations and evaluate NESOSPRAY HE-C under varying environmental conditions and for different rhinosinusitis etiologies.

Overall, the data from this study provide strong evidence of NESOSPRAY HE-C’s rapid onset and sustained relief of symptoms, supporting its potential as a safe, effective, and widely applicable non-pharmacological option for managing common cold and ARS.

## 5. Conclusions

NESOSPRAY HE-C provides rapid and sustained relief from the symptoms of acute rhinopharyngitis (common cold) and acute rhinosinusitis in children, pregnant women, and adults. As a safe and effective non-pharmacological option, it operates through purely mechanical mechanisms, including osmotic decongestion and the formation of a protective barrier. These actions make NESOSPRAY HE-C particularly valuable for patients who are contraindicated for or prefer to avoid systemic therapies.

## Figures and Tables

**Table 1:** Demographic statistics of the study population.

Variable	General	Group_R	Group_T	*p*-Value
Number of patients	55	26 (47%)	29 (53%)	X
Male (M)	M = 26 (47%)	M = 13 (23%)	M = 13 (24%)	0.91
Female (F)	F = 29 (53%)	F = 13 (24%)	F = 16 (29%)	0.91
Age (mean ± SD, [min, max])	23 ± 9.8 [6, 37]	22 ± 10.4 [6, 37]	23.9 ± 9.4 [6, 36]	0.59

**Table 2:** Demographic statistics of the pediatric population.

Variable	General	Group_R	Group_T	*p*-Value
Number of patients	19	10 (53%)	9 (47%)	X
Male (M)	M = 12 (63%)	M = 6 (31.5%)	M = 6 (31.5%)	1
Female (F)	F = 7 (37%)	F = 4 (21%)	F = 3 (16%)	1
Age (mean ± SD, [min, max])	10.6 ± 3.6 [6, 17]	9.9 ± 3.5 [6, 17]	11.4 ± 3.7 [6, 16]	0.36

**Table 3:** Demographic statistics of the pregnant women population.

Variable	General	Group_R	Group_T	*p*-Value
Number of patients	17	7 (41%)	10 (59%)	X
Sex (F)	F = 17 (100%)	F = 7(41%)	F = 10 (59%)	0.46
Age (mean ± SD, [min, max])	30.1 ± 3.2 [25, 35]	29.7 ± 2.4 [27, 33]	30.3 ± 3.7 [25, 35]	0.70

**Table 4:** Summary of statistical results for different days and treatments.

Symptoms	Baseline	After 2 h	Day 03	Day 06	Day 08
Rhinorrhea/Congestion	0.3557	0.0089	0.0030	1.38 × 10^−^⁸	7.55 × 10^−^⁹
Cough	0.2472	0.7872	0.0052	1.00 × 10^−^⁷	8.43 × 10^−^⁹
Sleep	0.0493	0.0493	0.0005	7.66 × 10^−^⁶	7.01 × 10^−^⁹
Facial Pain	0.7610	1.0000	0.0111	1.01 × 10^−^⁴	1.25 × 10^−^⁷
Fever	0.6719	0.3530	0.3923	1.40 × 10^−^⁴	3.12 × 10^−2^

**Table 5:** Global assessments by patients/parents and physicians on Day 8 or recovery.

Treatment	Very Much Improved	Much Improved	Minimally Improved	No Change	Minimally Worse	Much Worse	Very Much Worse
T	26	3	0	0	0	0	0
R	4	3	19	0	0	0	0

## Data Availability

The raw data supporting the conclusions of this article will be made available by the authors on request. The data are not publicly available due to privacy and institutional policy.
